# Intractable epilepsy due to angiocentric glioma: A case report and minireview

**DOI:** 10.3892/etm.2013.1402

**Published:** 2013-11-11

**Authors:** GUOQIANG CHEN, LIN WANG, JINTING WU, YONGJIAN JIN, XIAOSONG WANG, YULAN JIN

**Affiliations:** 1 Department of Neurosurgery, Xiangya Hospital, Central South University, Changsha, Hunan 410008, P.R. China; 2 Department of Neurosurgery, Yuquan Hospital, Tsinghua University, Beijing 100049, P.R. China; 3 Department of Pathology, Tongren Hospital, Capital Medical University, Beijing 100730, P.R. China

**Keywords:** angiocentric glioma, intractable epilepsy, cortical, electrocorticogram

## Abstract

The aim of this case report and minireview was to investigate the diagnosis of and therapeutic approaches for angiocentric glioma (AG) and to summarize the clinical manifestations and the pathological and imaging characteristics of the disease. Intraoperative cortical electroencephalogram (ECoG) monitoring was performed to locate the epileptic foci in a child with AG who presented with intractable epilepsy, prior to the total resection of the tumor being performed under the microscope. The clinical features, imaging characteristics, intraoperative conditions, surgical methods and pathological results were analyzed and compared with the literature. The review revealed that to date, the clinical features of the 52 reported cases of AG (including this case) have been mainly characterized by epilepsy. High T2-weighted image (WI) and fluid-attenuated inversion recovery (FLAIR) signals may be detected with magnetic resonance imaging (MRI) scanning of the cranium; however, no enhancement signals are detected by enhanced scanning. The prognosis following surgical resection is favorable. The lesions in the present case demonstrated clear boundaries with a central cystic affection accompanied by an arachnoid cyst on the left temporal pole. Pathological examination revealed that the lesion was positive for glial fibrillary acidic protein (GFAP), S-100 protein, vimentin, epithelial membrane antigen (EMA), cluster of differentiation 99 (CD99) and D2-40. The Ki-67/MIBk-1 labeling index was ~1%. In conclusion, AG exhibits characteristic features in imaging; however, its diagnosis depends on histopathological examination. The prognosis of total surgical resection is good and intraoperative ECoG may be used to assist positioning.

## Introduction

Angiocentric glioma (AG) is a rare type of tumor of the central nervous system (1). It was recognized as a tumor with distinct clinicopathological characteristics by the World Health Organization (WHO) in 2007 [International Classification of Diseases for Oncology (ICD-O) 9431/1, WHO level I]. The exact cause of this disease is unclear. The diagnosis of AG depends on tissue pathology, due to a lack of marked specific changes with brain computed tomography (CT). Magnetic resonance imaging (MRI) reveals a characteristic high T2-weighted image (WI) (2) and fluid-attenuated inversion recovery (FLAIR) signals (3,4), while enhanced scanning reveals no enhancement (5,6). According to clinical findings, most patients with AG suffer from intractable epilepsy. The majority of simple surgical excisions of the lesions result in a favorable prognosis, and postoperative pathology has indicated that most lesions are WHO level I.

A total of 52 cases of AG have been described in the literature (2,3,5–20), including the current case [three cases (21) have not been included in this total, due to a lack of specificity]. The majority of the case reports have focused on the imaging, histopathology and clinical manifestations of AG, while there has been less focus on studying the intraoperative conditions and methods for the surgical excision of the lesion. However, studies of this nature may be beneficial in establishing how to control the symptoms of epilepsy and are worthy of further investigation.

We report a case of seven-year-old female patient who, according to the preoperative presentation, appeared to be suffering from drug-refractory epilepsy. Lesions were located near the central sulcus in the right parietal cortex, and were concurrent with a left temporal pole arachnoid cyst. Intraoperative cortical electroencephalogram (ECoG) monitoring was used to assist with locating the epileptic foci, prior to the tumor and epileptogenic lesions being surgically resected. A postoperative study was conducted into the cytology and immunohistochemistry and the presence of AG (WHO level I) was demonstrated pathologically. The majority of the tumors described previously have been distinct entities; few have been combined with cystic degeneration and a simultaneously occurring left temporal arachnoid cyst. These are manifestations that cause a clinical concern for the neurosurgeon. We used intraoperative ECoG monitoring to locate the epileptic foci, prior to surgically resecting the tumor and epileptogenic lesions. This was beneficial for the control of postoperative epileptic seizures. This study may be used as a reference for clinical neurosurgeons. The case is described in the following sections.

## Case report

### General information

A seven-year-old girl presented at The Department of Neurosurgery, Yuquan Hospital, Tsinghua University (Beijing, China) with a three-year history of paroxysmal convulsions with loss of consciousness and a deterioration of the disease. The predominant forms of seizure were absence and whole body tonic-clonic seizures, while the main clinical presentation was a sudden bow (slightly forwards and downwards, to the left) in a single or continuous cluster of seizures. The body sometimes dipped slowly to the left and then to the right side while the upper and lower limbs stiffened, presenting absence seizures. The seizures were maintained for approximately half a minute (not >1 min), prior to remission. The administration of oral drugs, such as oxcarbazepine, clonazepam, topiramate and valproate, controlled the symptoms poorly and the attacks occurred 4–10 times monthly. There was no previous history of dystocia, hypoxia, encephalitis or any other medical history. Written informed consent was obtained from the patient’s family.

### Assisted examination

**Figure 1:**
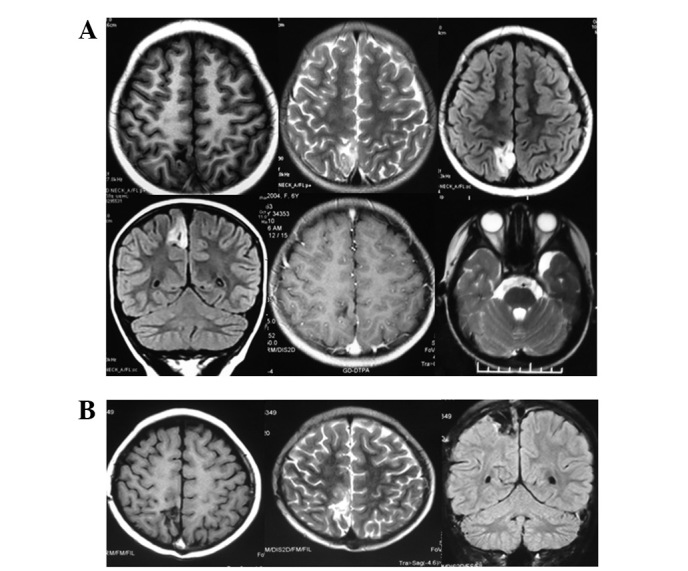
(A) Preoperative T1-weighted image (WI) showed a low signal, while fluid-attenuated inversion recovery (FLAIR) scanning showed a high signal and enhanced scanning revealed no signals. An arachnoid cyst was visible in the left temporal region. (B) At the six-month postoperative review there was no tumor recurrence.

Brain MRI imaging revealed a clearly visible boundary in the right parietal region of the cerebral falx and the cortex. It appeared as a wedge, with the cutting-edge pointing to the white matter of deep lesions. The T1-WI had a low signal (long T1), while the T2-WI had a high signal (long T2) and FLAIR imaging showed a high signal, with a low signal in the center. No enhancement signals were detected by enhanced scanning. A visibly widened subarachnoid cyst was observed in the left temporal pole. Postoperatively, it was considered that there was an embryonic dysplastic neuroepithelial tumor and left temporal pole arachnoid cyst (Fig. 1).

The various periods of the bilateral sleep-wake cycle led to a large number of high volatility waves. Video electroencephalogram (VEEG) showed 1.2–2.5 Hz sharp wave and sharp slow wave (distributing or continuously appearing), which were obvious in bilateral central and front temporal regions. The occurrence in right side was a little earlier than left side. During the process of monitoring, the seizures occurred several times and the clinical onset was predominantly a sudden bow (slightly forwards and downwards to the left). Seizures occurred singly or in continuous clusters. EEG concurrently displayed the highest conductivity of the high-amplitude biphasic slow waves or irregular slow waves on the right side.

### Surgery

**Figure 2:**
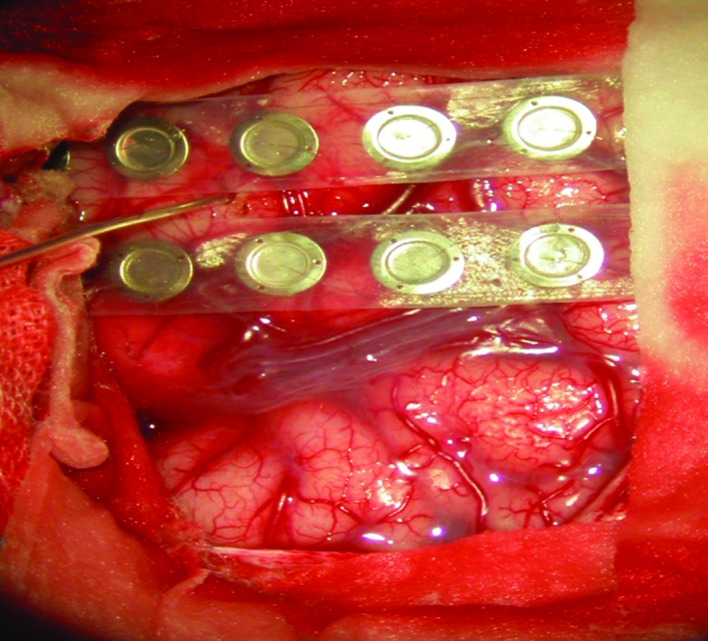
Intraoperative electroencephalogram EEG and deep cortical electroencephalogram (ECoG) monitoring.

Under general anesthesia, an incision was made in the right parietal lesions. During the surgery, local arachnoid thickening and subarachnoid widening were observed by cutting the dura mater. Intraoperative ECoG and deep EEG were monitored (Fig. 2).

The results demonstrated that local and deep spikes were emitted frequently and that fewer were recorded 1 cm away from the lesion edge. A few sharp wave and spike wave were visible in areas close to posterior part of precentral gyrus and postcentral gyrus. The lesions were located in cortical and subcortical regions; the size was ~2.5×3.0 cm and clear borders with the surrounding brain tissue were visible. Parts of the lesion were textured like a rotten fish and local central monitoring with the ECoG was repeated to reveal a small amount of spike-wave discharge in the central frontal gyrus on the midline at ~1 cm. There were no significantly abnormal EEGs monitored after using local cortical low power, bipolar coagulation and burning.

### Tissue pathology and the histopathological examination

The histological examination revealed that bipolar single cells in a monolayer or multilayers were centered around the cortical blood vessels in the form of ependymoma-like pseudorosettes along the vascular axis. The tumor cells were arranged in palisade-like structures in a swirling effect, giving the area of the tumor in the neural parenchyma a wide range of different cell densities. The nuclei of the tumor cells were elongated, the chromatin was finely granular, no or rare nuclear fission was present and no microvascular proliferation or necrosis was observed. There were no significantly heteromorphic neurons in the lesions.

**Figure 3:**
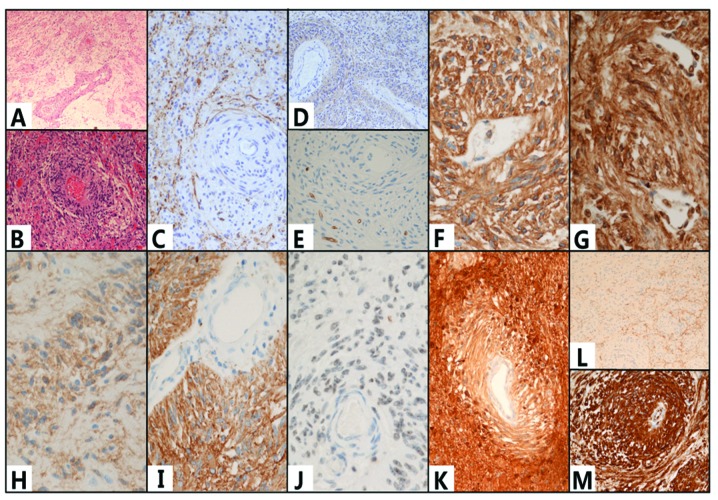
(A and B) Hematoxylin and eosin (H&E) staining showed that a large number of bipolar cells were clustered around and growing along the long axis of the the blood vessel. The cells were positive for human leukocyte differentiation antigen 34 (CD34) (E), CD99 (F) and D2-40 (G), glial fibrillary acidic protein (GFAP) (I), S-100 protein (K) and vimentin (M); however, they were negative for the neuron markers synaptophysin (Syn) (L) and chromaffin protein/NeuN. There was no expression of microtubule-associated protein 2 (MAP2) (C), nestin (D), epidermal growth factor receptor (EGFR) or (H) oligodendrocyte transcriptor-2 (Olig-2). The was expression of (J) epithelial membrane antigen (EMA) but no neurofilament (NF) in the tumor cells. (A) Magnification, ×10; (B–D,K,L) magnification, ×20; (E–J,M) magnification, ×40.

The tumor cells were positive for tumor cell glial fibrillary acidic protein (GFAP), S-100 protein, vimentin, human leukocyte differentiation antigen 34 (CD34), epithelial membrane antigen (EMA), cluster of differentiation 99 (CD99) and D2-40. However, the cells were negative for neuron markers, such as synaptophysin (Syn) and chromaffin protein/NeuN. There was no expression of oligodendrocyte transcriptor-2 (Olig-2), epidermal growth factor receptor (EGFR), neurofilament (NF), CD117, nestin or microtubule-associated protein 2 (MAP2) in the tumor cells. Furthermore, there were no visible changes in the p53 expression level. The Ki-67/MIBk-1 labeling index was ~1% (Fig. 3).

### Follow-up

The recovery of the patient was good following surgery and follow-ups were performed for 12 months. No nervous system dysfunction, such as epilepsy attacks, dizziness/headaches or hemiplegia, was observed and there were no abnormal somatosensory manifestations. MRI review did not reveal any residual tumor or tumor recurrence. No abnormal EEG signals were detected in the EEG review.

## Discussion

AG is a rare type of tumor with distinct clinicopathological characteristics. To date, there have been 52 cases of AG described in total (2,3,5–20), including the current case [three cases (21) have not been included in this total, due to a lack of specificity]. Morbidity due to AG may occur at any age and seizure is the main characteristic feature of the condition. The tumor develops slowly and may be cured by surgical resection.

A search of the PubMed index indicates that 52 cases of AG, including the current case, have been reported to date. The patients were aged between two (2) and 70 (6) years at the time of surgery and included: children (0–6 years old), 26.92% (14/52); juveniles (7–17 years old), 46.15% (24/52); young adults (18–46 years old), 21.15% (11/52); middle-aged adults (45–59 years old), 3.85% (2/52) and the elderly (aged >60 years), 1.92% (1/52). The course of the disease ranged from one week (23) to 57 years (6), while the male to female ratio of AG in the reported cases is 30:22. Therefore, it may be concluded that the majority of patients with AG and seizures are children and juveniles, with no significant differences in gender.

The combined analysis of the reported cases revealed that epilepsy was a predominant clinical manifestation for the majority of patients with AG, including 46 cases of intractable epilepsy (90.2%, 46/51; one of the studies (12) did not describe the symptoms and so was not included in the analysis). In 40 cases (8.43%, 40/51) the epilepsy manifested alone; in three cases (5.88%, 3/51) this was accompanied by symptoms of headache and dizziness; in two cases (17) (3.92%, 2/51) by symptoms of visual impairment and in one case (16) (1.96%, 1/51) by auditory hallucinations. In four cases (7.84%, 4/51) headache and dizziness manifested separately, while unsteady gait and hydrocephalus manifested separately in one case (11) (1.96%, 1/51). It has been revealed that epilepsy caused by a tumor accounts for ~17.8% of cases and that 20% of the tumors are AGs (21). AGs account for 2.3% of the cases of tumor-induced epilepsy in children (22).

MRI is beneficial when determining the location of the lesions and in making the diagnosis. Out of all described cases, MRI showed lesions in the frontal lobe in 11 cases (21.57%, 11/51); in the parietal lobe in seven cases (13.73%, 7/51); in the temporal lobe in 19 cases (37.25%, 19/51); in the occipital lobe in two cases (3.92%, 2/51) and in the hippocampus in four cases (7.84%, 4/51). Furthermore, there was one case (1.96%, 1/51) of a lesion in the occipitoparietal lobe, three cases (5.88%, 3/51) in the frontoparietal lobe, one case (1.96%, 1/51) in the frontotemporal lobe, one case (1.96%, 1/51) in the occipital temporal lobe, one case (1.96%, 1/51) in the thalamus (17) and one case (1.96%, 1/51) in the midbrain (11) in the junctional zone. The imaging shows that the tumors occurred mainly in the brain parenchyma and in each brain lobe. No coverage in the cerebral ventricle was observed. The common imaging features are that the brain tumor is typically located cortically or subcortically and forms wedge-shaped lesions, which diffusely infiltrate the surface in a stem-like (stalk-like) manner in the direction of the cerebral ventricle (1,4). The T2-WI and FLAIR imaging show high signals, while no enhancement signals are shown by enhanced scanning. (5,6). However, low (2) and high signals (3,4) may be identified on the T1-WI. The present case in our hospital showed a low signal T1-WI. At present, it is unclear whether the signal changes in the T1-WI are associated with bleeding (13) or calcification (4,17).

Although AG has been newly recognized as a distinct entity of tumor, compared with ependymoma (in particular, cortical or subcortical ependymoma), there are a number of similarities in clinical presentation and histopathology. However, AG has characteristic features, including mild visible atypia and local invasion of single and bipolar cells, as shown under the microscope. Furthermore, the cells of the tumor cluster around vessels and grow along the long axis of blood vessels in the network of nerve fibers. Immunohistochemistry has shown the tumor cells to be positive for GFAP (1), EMA (23), monoclonal antibody of cell proliferation-associated nuclear antigen (MIB-1) (10) and cell cycle-associated nuclear antigen Ki-67. In the present case, immunohistochemistry revealed that the tumor was positive for GFAP, S-100 protein, vimentin, CD34, CD99 and D2-40 and negative for neuron markers (Syn and chromaffin protein/NeuN). Furthermore, there was no expression of Olig-2, EGFR, NF, CD117 or MAP2 in the tumor cells and there were no visible changes in the p53 expression level. The Ki-67/MIBk-1 labeling index was ~1% (Fig. 3). The characteristic antibodies of glial and epithelial cells were expressed; however, the cells were negative for neuronal antibodies. In the current case there was no change in the expression of the proto-oncogene, p53, and the Ki-67/MIBk-1 labeling index demonstrated that the proliferation rate of the tumor was ~1%, which is consistent with the biological behavior of benign tumors.

AG, defined as WHO level I, has benign biological characteristics. The main purpose of therapy is to surgically excise the lesion and to control the seizures. Following the surgical excision of the lesions, the symptoms of epilepsy may be effectively controlled. Out of the 52 patients with AG, 34 patients underwent a complete surgical resection only (received surgery but not radiotherapy or chemotherapy) and 22 of the patients were followed-up for >12 months without suffering any seizures; nine patients underwent a partial resection (surgery plus radiotherapy and chemotherapy not included) and of the seven patients who received follow-ups for >12 months, two were seizure-free. These data show that full surgical resection of the tumor is more efficacious at controlling the symptoms of epilepsy. In the present case, intraoperative ECoG monitoring showed that the abnormal discharge range exceeded the area of the tumor, demonstrating that resection of the tumor alone does not completely eliminate the abnormal discharge. Since this may lead to epileptic seizures, surgical ECoG positioning may be used to determine the area requiring removal and to extend the target for resection, in order to ensure normal function. In the current study, low-power coagulation and cautery was performed, in order to obtain better clinical efficacy for the functional areas of the cortex that were unresected, but where abnormal discharge remained. This suggests that for AG, surgery and intraoperative stimulation, in the central area when necessary, are able to better ensure the efficacy of the surgery, with a smaller impact on the patient’s function. Previous studies have shown that in cases of AG with surrounding cortical dysplasia (17,19,21), intraoperative ECoG monitoring is necessary to ensure the complete resection of all of the epileptic foci, in addition to the tumor. In cases where the tumors have a deep location or when patients are unwilling to accept surgery, radiotherapy alone may be applied; however, it is necessary to initially biopsy the lesion, in order to confirm the pathological diagnosis.

The prognosis of the patients who have undergone total surgical resection for AG is typically good. The longest follow-up of patients with AG has reached 165 months, and the patients have been seizure-free in this period. Two patient mortalities have been reported: One patient (8) succumbed to bronchitis a week subsequent to surgery and one patient (7) succumbed to malignant tumors after 62 months. The slow growth rate of AG is generally <1%; but growth rates have been observed to reach 10% (14). It is increasingly being demonstrated that the cells in AG undergo active mitotic division and have a high proliferative capacity (18,20). Whether this may change the benign biological characteristics of the tumor remains inconclusive. However, the long-term follow-up for the outcome of this disease is very important.

The main clinical manifestation of AG is epilepsy. The imaging studies of AG exhibit clear features; however, a definite diagnosis depends on histopathological examination. The most effective treatment approach for the control of the epileptic seizures is to perform a total resection of the tumor and the area presenting with abnormal discharge using intraoperative ECoG positioning. Following the total resection to remove the lesions, the prognosis of the majority of the patients is favorable; however, a long-term follow-up of the patients after the surgery is required.
